# The complete chloroplast genome of *Dendrobium harveyanum* (Orchidaceae)

**DOI:** 10.1080/23802359.2019.1669088

**Published:** 2019-09-23

**Authors:** Zhi-Cong Huang, Yun-Yun Pan, Gui-Zhen Chen, Li Jun Chen, Xin-Yi Wu, Jie Huang

**Affiliations:** a Key Laboratory of National Forestry and Grassland Administration for Orchid Conservation and Utilization, Shenzhen, Guangdong, China;; b Shenzhen Key Laboratory for Orchid Conservation and Utilization, The National Orchid Conservation Centre of China and The Orchid Conservation and Research Centre of Shenzhen, Shenzhen, Guangdong, China

**Keywords:** *Dendrobium harveyanum*, chloroplast genome, phylogenetic, endangered species, Orchidaceae

## Abstract

*Dendrobium harveyanum* is an endangered species of Orchidaceae. Here we report the complete chloroplast (cp) genome sequence and the cp genome features of* D. harveyanum*. The complete cp genome sequence of *D. harveyanum* is 157,292 bp in length and presented a typical quadripartite structure including one large single-copy region (LSC, 86,583 bp), one small single-copy region (SSC, 19,449 bp), and two inverted repeat regions (IRs, 25,630 bp each). The cp genome encoded 138 genes, of which 120 were unique genes. The phylogenetic relationships show that *D. harveyanum* is closely related to other species in *Dendrobium*.

The genus *Dendrobium* belongs to the tribe Dendrobieae (Orchidaceae: Epidendroideae) and is one of the three largest genera in the family Orchidaceae with ∼1500 species, ranging through India across to Japan, south to Malaysia, and east to Australia, New Guinea, and the Pacific islands and is the second most common orchid genera in cultivation after the *Cattleyas* (Chen et al. 2009; Pridgeon et al. 2014). Since the establishment of *Dendrobium* by Swartz (Swartz 1799), various generic delimitations and infrageneric systems have been proposed (Li et al. 2005; Xu et al. 2014; Wang et al. 2017). *Dendrobium* species have an enormous economic value in global horticultural trade (Teixeira da Silva et al. 2016), and some *Dendrobium* species also have medicinal and pharmaceutical values (Teixeira da Silva et al. 2015). Genomic studies may contribute to the study of species identification, germplasm diversity, and genetic engineering (Lin et al. 2015). The complete chloroplast (cp) genome sequence of *D. harveyanum* was assembled in this study.

Leaf samples of *D. harveyanum* were obtained from the Orchid Conservation and Research Centre of Shenzhen and specimens were deposited in the National Orchid Conservation Center herbarium (NOCC; specimen code Z.J.Liu 2509). Total genomic DNA was extracted from fresh material using the modified CTAB procedure of Doyle and Doyle (1987), and sequenced on Illumina Hiseq 2500 platform (San Diego, CA). Genome sequences were screened out and assembled with MITObim v1.8 (Hahn et al. 2013), which resulted in a complete circular sequence of 157,292 bp in length. The cp-genome was annotated with CpGAVAS (Liu et al. 2012).

The cp genome sequence of *D. harveyanum* (MN245570) is 157,292 bp long and presented a typical quadripartite structure including one large single-copy region (LSC, 85,583 bp), one small single-copy region (SSC, 19,449 bp), and two inverted repeat regions (IRs, 25,630 bp each). The cp genome encoded 138 genes, of which 120 were unique genes (85 protein-coding genes, 31 tRNAs, and 4 rRNAs).

Thirty-eight accessions of *Dendrobium* and two outgroups were used for the molecular analysis. The phylogenetic tree was constructed based on the maximum-likelihood (ML) method. The ML analysis was performed using the CIPRES Science Gateway web server (RAxML-HPC2 on XSEDE 8.2.10) with 1000 bootstrap replicates and settings as described by Stamatakis et al. (2008). The result showed that they were all clustered together (Figure 1). This newly reported cp genome will be helpful for further study on phylogenetic study, species identification, and genetic engineering.

**Figure 1.**
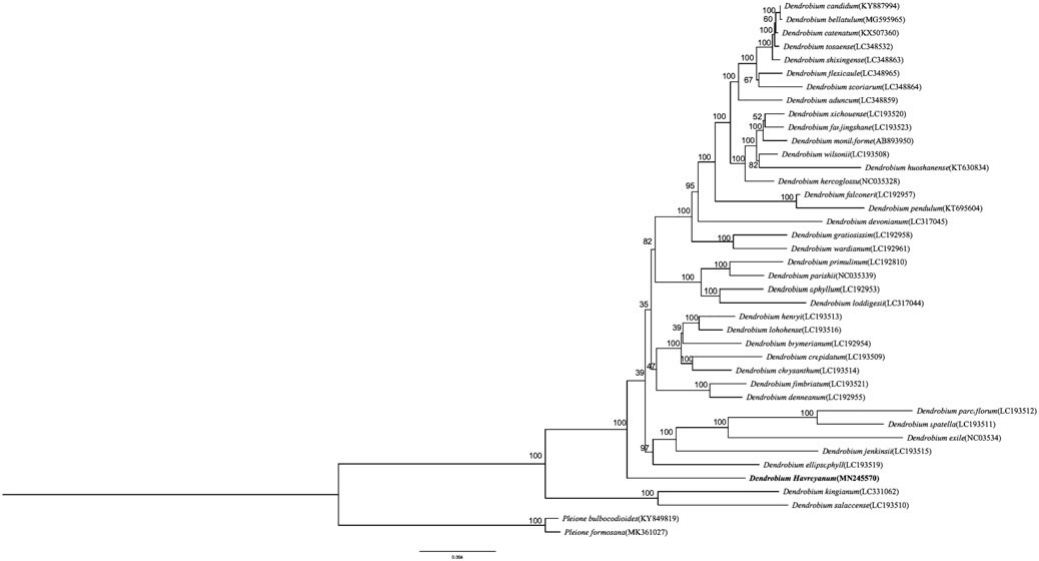
Phylogenetic position of *Dendrobium harveyanum* inferred by maximum-likelihood (ML) of complete cp genome. The bootstrap values are shown next to the nodes.
